# Das prozessierte EEG zur personalisierten Dosierung von Anästhetika während Allgemeinanästhesie

**DOI:** 10.1007/s00101-023-01313-0

**Published:** 2023-08-08

**Authors:** F. Lersch, T. J. G. Zingg, J. Knapp, F. Stüber, D. Hight, H. A. Kaiser

**Affiliations:** 1 Universitätsklinik für Anästhesiologie und Schmerzmedizin, Inselspital, Universitätsspital Bern, Universität Bern, Freiburgstrasse, 3010 Bern, Schweiz; 2 Zentrum für Anästhesiologie und Intensivmedizin, Hirslanden Klinik Aarau, Hirslanden AG, Schänisweg, 5001 Aarau, Schweiz

**Keywords:** Elektroenzephalogramm, Allgemeinanästhesie, Koma, Intraoperatives neurophysiologisches Monitoring, Dosis-Wirkungs-Beziehung, Hypnotika, Analgesie, Electrencephalogram (EEG), Anesthesia, general, Coma, Intraoperative neurophysiological monitoring, Dose response relationship, Hypnotic drugs, Analgesia drugs

## Abstract

Die Elektroenzephalogramm(EEG)-gesteuerte Anästhesie ist aus modernen Operationssälen nicht mehr wegzudenken und hat sich als Standard-Monitoring etabliert. Viele Anästhesisten verlassen sich dabei auf die prozessierten EEG-Indizes und hoffen, dadurch ihren Patienten anästhesiebedingte Komplikationen wie intraoperative Awareness, postoperatives Delir oder andere kognitive Komplikationen zu ersparen. Diese Übersichtsarbeit fasst klinisch relevante Informationen und Studien über die 5 im Klinikalltag am häufigsten verwendeten Anästhesietiefe-Monitore zusammen. Messprinzipien, die den verschiedenen Monitoren zugrunde liegen, werden erläutert. Zudem wird eine praktische Anleitung im Umgang mit potenziellen Artefakten und irreführenden „Trendanzeigen“ im prozessiertem EEG angeboten.

## Abstract (en)

Electroencephalogram (EEG)-guided anesthesia is indispensable in modern operating rooms and has become established as the standard form of monitoring. Many anesthesiologists rely on processed EEG indices in the hope of averting anesthesia-related complications, such as intraoperative awareness, postoperative delirium and other cognitive complications in their patients. This educational review aims to provide information on the five most prevalent monitors used to guide depth of sedation during general anesthesia. This article elucidates the principles underpinning the application of these monitors where known, which are generally based on power in various EEG frequency bands and on the burst suppression pattern. Convinced that EEG-guided anesthesia has the potential of benefitting many surgical patients, it is felt that many basic principles and shortcomings of processed EEG indices need to be better understood in the clinical practice. After discussing the different monitors and clinically relevant data from the literature, the article gives a short practical guidance on how to critically interpret processed EEG information and troubleshooting of confounded indices in the context of clinical situations.

## Einführung

Die intraoperative Awareness (unerwünschter Wachzustand) während einer Anästhesie ist eine von Patienten und Anästhesisten gefürchtete Komplikation. Hierbei können sich Patienten an Situationen während einer Operation erinnern, die von Unterhaltungen des Personals bis hin zu schmerzhaften Zuständen reichen. Ein erhöhtes Awareness-Risiko wurde bei Kaiserschnitten in Allgemeinanästhesie, bei herzchirurgischen Eingriffen, bei Gebrauch von Muskelrelaxanzien, bei polymorbiden Patienten, bei vorbestehendem Drogenabusus, bei totaler intravenöser Anästhesie (TIVA) und bei Notfalloperationen beschrieben [2]. In den vergangenen Jahrzehnten haben sich zur Vermeidung solcher Zwischenfälle verschiedene Elektroenzephalogramm(EEG)-basierte Monitore etabliert, welche aus unterschiedlichen Komponenten des EEG einen Zahlenwert („Index“) als Äquivalent für die „Anästhesietiefe“^1^ berechnen (prozessiertes EEG, pEEG). Der Wert des Index liegt in der Regel zwischen 0 und 100, wobei 0 einem tiefen Koma und 100 dem Wachzustand entspricht.

Die Messung der „Anästhesietiefe“ wird seit Jahren kontrovers diskutiert, da die Allgemeinanästhesie aus der Kombination von Hypnose, Analgesie, Amnesie und Immobilität definiert ist [3], die pEEG-Monitore aber lediglich die hypnotische Komponente erfassen, auch wenn Schmerzreize diese Ebene bei unzureichender Analgesie beeinflussen können. Der Bewusstseinsverlust kann dennoch als zentrale Bedingung für eine Allgemeinanästhesie betrachtet werden, und mit adäquater Dosierung der Anästhetika sollte es möglich sein, eine intraoperative Awareness zu verhindern [4]. Das Konzept der „Anästhesietiefe“ impliziert kontinuierliches Durchschreiten verschiedener Stadien des Bewusstseinsverlustes bei steigender Anästhetikakonzentration im Gehirn. Diese Stadien beinhalten den Verlust der Gedächtnisbildung, einer Reizantwort, des Bewusstseins sowie von unwillkürlichen Bewegungen auf einen chirurgischen Reiz [5]. Mit den aktuellen Anästhetika und Muskelrelaxanzien haben die klassischen, klinischen Stadien der „Anästhesietiefe“ nach Guedel an Bedeutung verloren [6]. Da zudem Bewegungen oder vegetative Reaktionen auf einen chirurgischen Reiz meist auf spinaler Ebene vermittelt werden [7, 8], ist die Nutzung eines verlässlichen EEG-Monitors zur Beurteilung der Wirkung von Anästhetika auf das Gehirn sinnvoll. Bereits 1937 konnten während einer Anästhesie mit Chloroform spezifische EEG-Veränderungen beobachtet werden [9], weshalb das EEG schon damals zur Bestimmung der „Anästhesietiefe“ vorgeschlagen wurde [10]. Das Grundprinzip des pEEGs beruht auf der Messung von Verschiebungen der Frequenzanteile des frontalen Roh-EEGs zu langsameren Frequenzen (von β zu α und δ), welche bei zunehmenden Konzentrationen an GABAergen Anästhetika auftreten (GABA: γ‑Aminobuttersäure, [11]). Für Details hierzu: s. Teil 1 dieses Beitrags (CME-Artikel, [1]).

Gewisse pEEG-Monitore leiten aus den jeweils vorherrschenden Frequenzen im Roh-EEG einen Index ab, der die „Anästhesietiefe“ widerspiegeln und die thalamische Hyperpolarisation sowie kortikale Synchronisation repräsentieren soll [12]. Andere Monitore wiederum bestimmen lediglich das „Ausmaß der Unordnung“ (Entropie) im EEG [13]. Die Mehrzahl der Algorithmen unterliegt jedoch dem Produktschutz und ist nicht im Detail publiziert.

**Figure 1:**
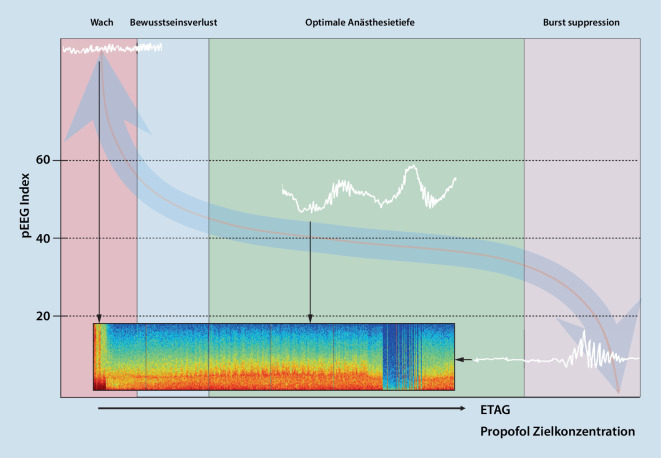

Eine Korrelation zwischen pEEG-Indizes, Anästhetikakonzentrationen im Gehirn und der klinischen Einschätzungen der Sedationstiefe ist eine essenzielle Voraussetzung für ein verlässliches „Anästhesie- bzw. Sedationstiefe“-Monitoring [14]. Diverse Studien zeigen, dass die meisten pEEG-Indizes Trends der Anästhetikakonzentrationen (von volatilen Anästhetika und Propofol) im Effektkompartiment Gehirn (Ziel- oder Wirkort entsprechend) während der Ein- und Ausleitung einer Allgemeinanästhesie gut abbilden können [15–17]. Eine ideale Beziehung von Anästhetikakonzentration zu pEEG-Indizes würde hierbei ein lineares Verhältnis darstellen. Die typische Dosis-Index-Kurve (Anästhetikakonzentration gegen pEEG-Index aufgetragen) ist jedoch sigmoidal, mit einer meist ausgeprägten Plateauphase der Indizes jenseits des Bewusstseinsverlustes über weite Dosisbereiche der Anästhetika (Abb. 1; [17–22]). Der Zielbereich des pEEG-Index für eine Allgemeinanästhesie befindet sich dabei üblicherweise zwischen den zwei klinischen Endpunkten *Bewusstseinsverlust* (oberflächliche Anästhesie/Risiko einer intraoperativen Awareness) und *Burst Suppression* (zu tiefe Anästhesie/evtl. erhöhte Morbidität und Mortalität).

In der Plateauphase sind Dosisveränderungen der Anästhetika durch alleinige Betrachtung des pEEG-Index kaum zu erkennen. Im Roh-EEG und im spektralen EEG sind sie hingegen gut nachvollziehbar. Darum ist von einer zu geringen Sensitivität der pEEG-Monitore für diese Dosis- bzw. EEG-Veränderungen auszugehen [23]. Der Bewusstseinsverlust bzw. das Wiedererlangen des Bewusstseins befindet sich auf dem linken steilen Abschnitt der Kurve bzw. am linken Rand des Plateaus, in welchem kleinere Veränderungen der Anästhetikakonzentration eine große Veränderung des pEEG-Index verursachen. Im klinischen Alltag kann es somit in diesem Übergangsbereich nach nur minimaler Veränderung der Anästhetikazielkonzentration zu unerwarteten Aufwachreaktionen von Patienten kommen. Am anderen Ende des Spektrums ergibt sich das Risiko der Burst Suppression, in dessen Bereich es zu einem steilen Abfall des Index kommt. Zudem besteht eine hohe interindividuelle Variabilität des Verhältnisses zwischen Wirkortkonzentration und pEEG-Index [19]. Dies erschwert die Festlegung eines Standardzielbereiches der pEEG-Indizes, der für alle Patienten gültig ist. Die verschiedenen pEEG-Indizes basieren zudem auf unterschiedlichen EEG-Parametern und Algorithmen. Zum besseren Verständnis des prozessierten EEG werden daher im folgenden Artikel die Datenlage und die Messmethodik der 5 momentan gängigsten Monitore der „Anästhesietiefen“-Messung erläutert und auf Fallstricke bei der Interpretation hingewiesen. In Tab. 1 werden allgemeine Eckdaten der verschiedenen Monitore zusammengefasst.

**Table 1:** 

	Jahr der Markteinführung	Anzahl der Studien	Kosten/Monitor	Kosten/Anwendung	Referenzwert für Allgemeinanästhesie	Algorithmus
*BIS*	1996	3240	Ca. € 4500	€ 10–15	40–60	BSR, Aktivität im niedrigen Gamma-Bereich (40–47 Hz)
*Narcotrend*	2000	131	Ca. € 23.000	€ 1–3	Ebene D2–D0 bzw. Index 37–64	Schlafstadien nach Loomis (1937, [47])
*Entropie*	2004	39 RE 104 SE	Ca. € 6500	€ 10	40–60	Mass an Entropie (Unordnung) des EEG
*Sedline*	2000	69	k. A.	€ 15	25–50	Prädiktives Modell (Power, Kohärenz)
*qCON*	2013	12	k. A.	€ 20–25	40–60 qNOX 0–20	Neural Network Regression

*BSR* Burst Suppression Ratio, *RE* „Response“-Entropie, *SE* „State“-Entropie, *k.* *A.* keine Angaben

## Kurzkasuistik

### Fallvignette, Teil 1

Ein 51-jähriger Patient mit langjährigem Alkoholabusus und ausgeprägter Wernicke-Enzephalopathie stellt sich zur Durchführung einer beidseitigen Kataraktoperation vor. Der Patient hat große Angst vor der OP und war im Rahmen seiner Alkoholabhängigkeit bei jeder vorherigen Anästhesie gewalttätig delirant gewesen. Teilweise waren die Delirepisoden durch Grand-Mal-Epilepsien geprägt. Die Enzephalopathie des Patienten geht mit einer Vielzahl nichtkontrollierbarer motorischer Bewegungen einher. Dieser Umstand gibt den Ausschlag, mit ihm und der betreuenden Ehefrau trotz erhöhtem Delirrisiko eine EEG-gesteuerte, multimodale, opioidsparende Kombinationsanästhesie (Allgemeinanästhesie mittels TIVA + Subtenonregionalanästhesie) zu vereinbaren.

**Figure 2:**
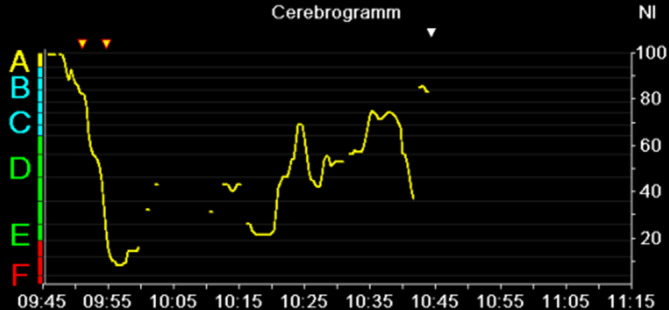

Nach Prämedikation mit Clonidin 150 µg p.o. und 6 mg Melatonin p.o. erhielt der in Begleitung seiner Gattin in die Anästhesievorbereitung kommende Patient 16 µg Dexmedetomidin i.v. Das Standard-Anästhesie-Monitoring wurde um ein frontales 3‑Elektroden-EEG (Narcotrend®, Narcotrend-Gruppe, Hannover) erweitert. Während der Vorbereitungen für die Anästhesie hielt der Patient die Hand seiner Ehefrau und hörte selbstgewählte beruhigende Musik. Einleitung und Einlage einer Larynxmaske erfolgten problemlos nach Gabe von Propofol und Alfentanil i.v. Das Auge mit dem ausgeprägteren Befund wurde nach Gabe eines Ketaminbolus von 0,5 mg/kgKG mittels Subtenonblock regional anästhesiert. Der pEEG-Index (von Narcotrend®, Abb. 2) zeigte nach Einleitung der Anästhesie einen steilen Abfall von über 90 (Wachzustand) auf unter 20 (Burst Suppression entsprechend) und stieg nach Gabe eines Ketaminbolus wieder auf über 60 an.

Der intraoperative Verlauf gestaltete sich, abgesehen von einem kurzen hypertensiven Abschnitt, während der Burst-Suppression-Phase ereignislos. Zum eigenen Erstaunen und dem seiner Ehefrau war der Patient nach Entfernung der Larynxmaske in „tiefer“ Sedation und halbstündigem Schlaf ruhig und in allen Ebenen orientiert und konnte nach 2 h ohne Auftreten eines Delirs ins häusliche Umfeld entlassen werden.

Um zu erläutern, dass bei dem beschriebenen Fall eine ausreichende „Anästhesietiefe“ vorhanden war, trotz nichtmessbaren und teils zu hohen pEEG-Indizes, werden die häufigsten pEEG-Monitore mit ihren Vor- und Nachteilen vorgestellt. Darauffolgend werden wir in einem zweiten Teil die Fallvorstellung mit Kommentaren und Interpretationen zu EEG und deliriumfreiem Verlauf ergänzen.

## Spezifische technische und evidenzbasierte Unterschiede prozessierter EEG-Monitore

### Bispectral Index™ (BIS™, Medtronic, Dublin, Irland)

**Figure 3:**
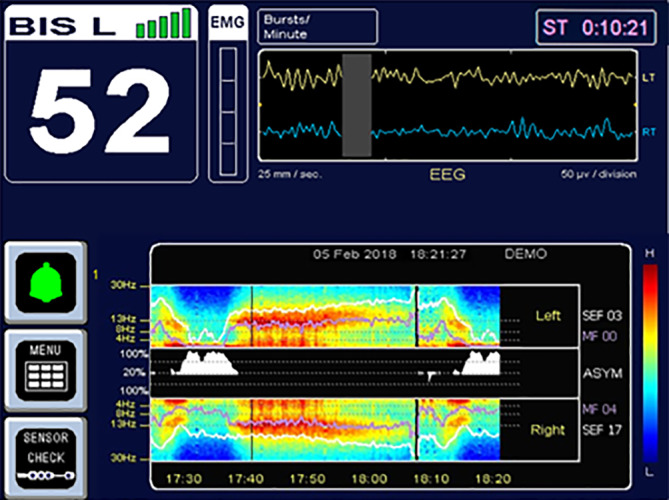

Der BIS™-Monitor (Abb. 3) wurde 1996 zur klinischen Verwendung zugelassen und hat sich seither zum Marktführer, betreffend des „Anästhesietiefe“-Monitorings, etabliert. Das EEG hierfür wird mit einem gerätespezifischen Einwegelektrodenband abgeleitet, welches in 2 Versionen zu uni- oder bilateraler Anwendung erhältlich ist. Die empfohlenen BIS-Werte während einer Allgemeinanästhesie liegen zwischen 40 und 60. Bis vor kurzem war das Anzeigen eines Spektrogramms nur während der Messung mit den bilateralen Sensoren möglich, nun ist dies auch für den unilateralen „alten“ Sensor verfügbar. Der BIS™-Monitor ist mit derzeit 3240 Zitationen auf PubMed der am meisten untersuchte und bekannteste Monitor zur Messung der „Anästhesietiefe“.

Der BIS-Index zeigt Werte zwischen 0 (Koma) und 100 (wach) an und wird aus 2 Komponenten des EEG aus den zurückliegenden 60 s berechnet. Es wurde angenommen, dass dem BIS™-Monitor zur Generierung des Index bispektrale Analysen des EEG zugrunde liegen [24]. Hierbei wurden die folgenden EEG-Auswertungsverfahren als wesentliche Komponenten des BIS-Algorithmus diskutiert:

1. die „*relative β‑Ratio*“ als Anteil an höheren Frequenzen (> 30 Hz) im Roh-EEG,
2. die *Sync-Fast-Slow *als ein Maß der Phasenkopplung neuronaler Oszillationen als mutmaßlicher Parameter für die Kommunikation zwischen verschiedenen Hirnarealen,
3. die Burst Suppression Ratio (BSR) [25].

Nun wurde aber von Connor gezeigt, dass der BIS-Algorithmus v. a. durch Aktivität im Frequenzbereich zwischen 40 und 47 Hz sowie das Vorkommen von Burst Suppression berechnet wird [26]. Dies erklärt, warum der BIS-Algorithmus bzw. -Index so ausgeprägt auf Muskelaktivität ansteigt und dementsprechend auf die Gabe von Muskelrelaxanzien abfällt [27]. BSR-Werte > 40 % sind linear mit BIS-Werten von 30 bis 0 korreliert; bei BSR-Werten unter 40 % ergab sich keine Korrelation [28].

Vom britischen „National Institute for Health Research“ [29] wurde 2013 in einer detaillierten Analyse gezeigt, dass die Verwendung des BIS™-Monitors bei Patienten mit erhöhtem Risiko für eine intraoperative Awareness unter TIVA kosteneffizienter als die klinische Standardüberwachung ist. Im Gegensatz dazu fiel die *Kosteneffizienz* in der allgemeinchirurgischen Population (ohne erhöhtes Awareness-Risiko) bei TIVA wesentlich geringer aus. Auch bei Inhalationsanästhesie war die Kosteneinsparung geringer als bei einer TIVA.

In Anbetracht der sigmoidalen Dosis-Index-Kurve sowie des Einflusses der EMG-Aktivität auf den BIS-Algorithmus verwundert es nicht, dass die Datenlage zur Senkung der Inzidenz von intraoperativer *Awareness* aus randomisierten Studien widersprüchlich ist. Ekman et al. [30] und Myles et al. [31] konnten eine Reduktion an intraoperativer Awareness in der BIS-gesteuerten Gruppe zeigen. Hierbei verglich Ekman seine BIS-geführte Gruppe zu einer historischen Kontrollgruppe und Myles zu einer Gruppe unter Standardtherapie. Beide Gruppen wiesen einen TIVA-Anteil von 40 % auf. Dieser Nutzen konnte jedoch in der Studie von Avidan et al. 2008 [32] nicht reproduziert werden, wobei in der nicht-BIS-gesteuerten Patientengruppe ein Alarm für die untere Grenze der endtidalen minimalen alveolären Konzentration (MAC) von 0,6 gesetzt wurde. In der daraufhin multizentrisch durchgeführten Studie mit über 6000 Patienten („BAG-RECALL trial“) ergaben sich in der Gruppe, die mit endtidalem MAC-Alarm geführt wurde, sogar weniger intraoperative Awareness-Fälle als in der BIS-gesteuerten Gruppe [33]. Die größte Studie zu intraoperativer Awareness (*n* = 21.601) in einer allgemeinen chirurgischen Population mit volatilen Anästhetika zeigte: Kein signifikanter Unterschied zwischen Narkoseführung mit BIS-Monitoring und endtidalem MAC-Alarm, dagegen signifikant mehr intraoperative Awareness-Fälle in der Gruppe ohne die genannten Intervention bzw. Vorgaben zur Anästhesieführung [34, 35]. Ein Vorteil hinsichtlich der Inzidenz intraoperativer Awareness durch die Verwendung des BIS™-Monitors zeigt sich konsistent in Studien mit einem höheren Anteil an TIVA [31, 33, 36]. Nicht näher untersucht wurde bisher, ob die Nutzung und Interpretation des spektralen EEGs zur Senkung der intraoperativen Awareness beitragen könnte [37]. Dies setzt in der Praxis aber voraus, dass anästhesieführende Kliniker EEG-Informationen ins klinische Bild einbeziehen können und pharmakotherapeutische Entscheidungen daraus ableiten.

Auch beim Thema *postoperatives Delirium* ist die Studienlage alles andere als eindeutig: Nachdem v. a. retrospektive Studien und zahlreiche kleinere randomisierte kontrollierte Studien [38] bei intraoperativer Verwendung eines BIS™-Monitors zur Titrierung der Anästhetika eine geringere Inzidenz an postoperativem Delir gezeigt haben, konnte dies in der großen, randomisierten ENGAGES-Studie (*n* = 1232) nicht bestätigt werden [39]. Allerdings lag auch in der BIS-Gruppe die kumulative Zeit der Burst Suppression bei 7 min im Vergleich zu 13 min in der Kotrollgruppe. Dies spiegelt sich auch in einem geringen Unterschied der endtidalen MAC-Werte wider (0,69 vs. 0,8). Interessanterweise hatte die Studie als Nebenresultat gezeigt, dass in der EEG-geführten Gruppe die *Mortalität* und der Vasopressorenverbrauch niedriger waren. Nach Publikation ergaben sich hierzu außergewöhnlich viele Kommentare in diversen Journalen. Auch eine retrospektive Analyse von 24.000 Allgemeinanästhesien wies einen Zusammenhang zwischen dem „Triple-low“-Zustand (Kombination aus niedrigem MAC < 0,8, niedrigem BIS < 45 und niedrigem mittleren Blutdruck < 75 mm Hg) und der postoperativen 30-Tage Mortalität nach [40]. Das entsprechende Editorial „Murderer, mediator, or mirror“ stellt aber die Kausalität gekonnt infrage [41]. Auch eine randomisierte kontrollierte Studie mit 6600 Patienten konnte keinen Vorteil hinsichtlich der Einjahresmortalität bei Patienten nachweisen, bei denen während der Operation ein BIS-Index von 35 bzw. 50 angestrebt wurde [42]. Weitgehend unbestritten ist jedoch, dass die Verwendung des BIS™-Monitors zur Steuerung der Anästhesie die verabreichte Menge an Anästhetika reduzieren lässt und zu einer schnelleren Erholung der Reaktionsfähigkeit und *Entlassung aus dem Aufwachraum* führt [43–45].

### Narcotrend®-Compact M (Narcotrend®, Narcotrend-Gruppe, Hannover, Deutschland)

**Figure 4:**
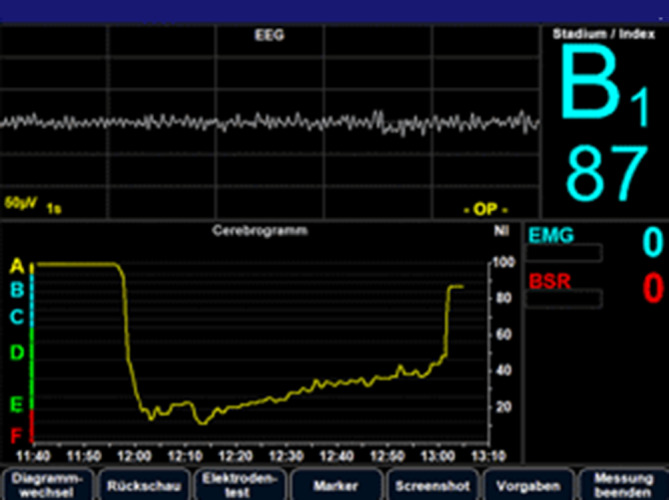

Der Narcotrend®-Monitor (Abb. 4) ist neben dem BIS-Verfahren eines der ältesten „Anästhesietiefe“-Monitorsysteme und wurde im Jahre 2000 eingeführt [46]. Die Literatursuche nach den Stichwörtern „Narcotrend“, „Narcotrend Index“ und „Narcotrend monitor“ zeigt in PubMed 137, 90 bzw. 119 Publikationen. Der Preis für eine Compact-M-Ausführung des Narcotrend®-Monitors liegt bei rund 23.000 €. Diesen vergleichsweisen hohen Anschaffungskosten stehen niedrige Elektrodenkosten im täglichen Gebrauch gegenüber, weil handelsübliche EKG-Elektroden eingesetzt werden können. Eine Kosten-Effektivität-Analyse wertete den Narcotrend®-Monitor als *günstiger* im Vergleich zu BIS- und Entropie-Monitoring (s. unten) [29], wobei aber bisher keine großen randomisierten Studien zu Delirium oder intraoperativer Awareness mit ihm durchgeführt wurden.

Gegenüber der Darstellung verschiedener „Anästhesietiefen“ als numerische Werte wie im BIS™-Monitor teilen die Algorithmen des Narcotrend-Monitors^→^ die „Anästhesietiefe“ in Stadien ein, die durch eine Kombination aus Kennbuchstaben und Zahl angezeigt werden. Diesen Stadien liegen Arbeiten aus frühen Tagen der Schlafforschung zugrunde. Loomis teilte 1937 die ineinander übergehenden Schlafstadien wie folgt ein [47]: „*A*“: wach mit langsamen Augenbewegungen, „*B*“: somnolent, leichtes Schlafstadium (heute Non-REM1 (Rapid Eye Movement)), niedrige EEG-Voltage und mittlere Frequenz, „*C*“ und „*D*“ bei Auftreten von Schlafspindeln, „E“ bei Verdrängung dieser Spindeln durch Deltawellen, und „*F*“ reflektiert das Auftreten von Burst Suppression und zunehmender Isoelektrizität, die im gesunden Schlaf nie auftreten. Dieser veranschaulichende Vergleich von Roh-EEG-Motiven und „Anästhesietiefe“-Stadien stellt eine der didaktischen Stärken des Gerätes dar. Parallel hierzu wird ein Narcotrend^®^-Index ausgegeben, welcher wie beim BIS™-Monitor von 0 bis 100 reicht, der angegebene Zielbereich während Allgemeinanästhesie liegt zwischen 37 und 64 (entspricht D2 bis D0).

Die aktuelle Ausführung des Narcotrend®-Monitors lässt neben einem frontalen Ein-Kanal-EEG aus 3 Elektroden auch ein mehr frontoparietales Zwei-Kanal-EEG aus 4 oder 5 Elektroden zu. Beim Zwei-Kanal-EEG kann durch den Vergleich der Alpha- und Beta-Leistung beider Hirnhemisphären ein *Surrogatmarker für einseitige Hypoperfusion* abgeleitet werden. Allerdings ist die klinische Relevanz bisher nie systematisch überprüft worden. Dass EEG-Veränderungen kritische Minderperfusion während der Karotischirurgie abbilden können, wurde schon 1987 durch Messick et al. gezeigt und ist reproduzierbar [48]. Verschiedene erfolgreiche Ansätze zur Verwendung des quantitativen EEG im Screening zerebraler Ischämien als intraoperatives Monitoring während Karotis- oder Herzchirurgie wurden in einer Übersichtsarbeit zusammengefasst [49]. Aufgrund der klinisch üblichen frontalen Montage des perioperativer EEG entgehen uns aber z. B. Ischämien des Mediastromgebietes.

Der Narcotrend®-Monitor wurde wiederholt mit dem BIS-Monitoring verglichen und zeigte vergleichbaren Nutzen bei den klinischen Endpunkten *Anästhetikaverbrauch* oder *Aufenthaltszeit im Aufwachraum* [50]. Alter scheint zum größten Teil keinen Einfluss auf die Berechnung des Narcotrend®-Index zu haben [51, 52], wobei grundsätzlich die atrophiebedingten EEG-Veränderungen betagter Patienten als auch sich wandelnde EEG-Signaturen während der Hirnreifung bei Kleinkindern unter 2 Jahren mögliche Fehlerquellen für die Berechnung des Index darstellen können [53].

### Patient State Index (PSI von SedLine® Masimo, Irvine, CA, USA)

**Figure 5:**
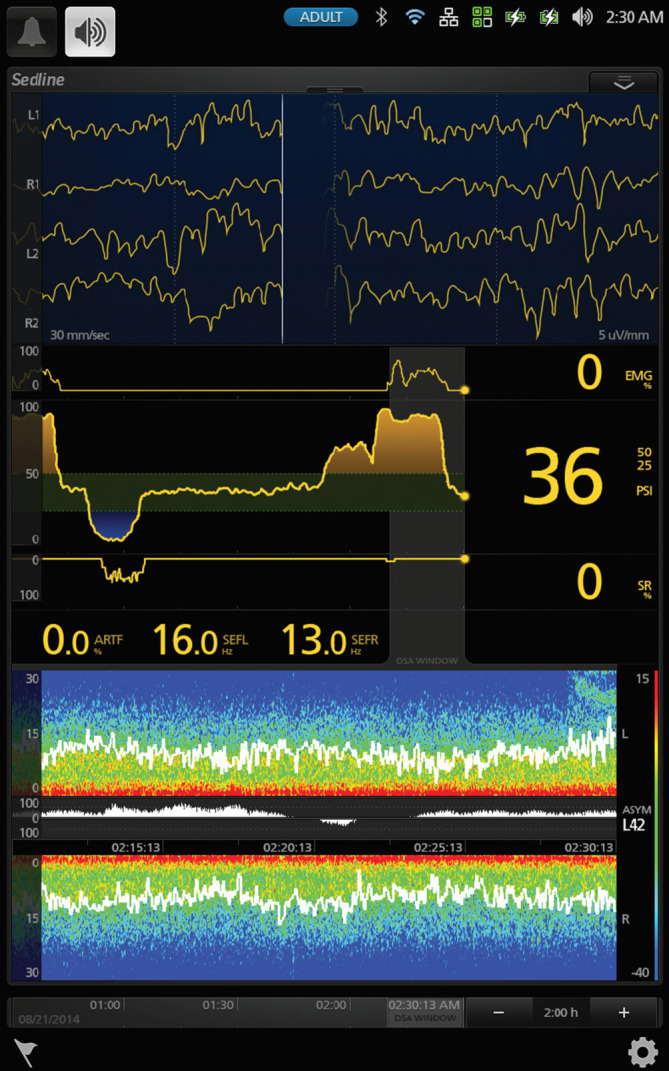

Der Sedline®-Monitor (Abb. 5) wurde im Jahr 2000 eingeführt [54] und im selben Jahr für den Gebrauch bei Allgemeinanästhesie zugelassen [55]. Die Publikationen zum Zeitpunkt der Markteinführung erwähnten, dass der generierte Patient State Index (PSI) aus frontalen, zentralen und posterioren EEG-Ableitungen berechnet wird [56]. Es muss aber angenommen werden, dass der Algorithmus gegenwärtig nur frontale Parameter einschließt, da das Sedline®-Monitoring keine zentralen oder posterioren Elektroden beinhaltet. Das gerätespezifische Einwegelektrodenband wird frontal geklebt und registriert 4 EEG-Kanäle. Die Literaturrecherche in PubMed ergibt 69 Publikationen mit den Begriffen „Patient State Index“ oder „Sedline“ im Titel oder Abstract. Gemäß Hersteller liegen die optimalen Indexwerte für eine Allgemeinanästhesie im Vergleich zu anderen kommerziellen Monitoren deutlich niedriger: zwischen 25 und 50.

Der ursprüngliche dem PSI zugrunde liegende Algorithmus wurde aus einem Datensatz von 176 EEG-Aufnahmen während Allgemeinanästhesie mit Propofol, volatilen Anästhetika und/oder Lachgas entwickelt [11]. Die genauen EEG-Parameter für die Berechnung sind unbekannt, werden aber als „Power“- und „Kohärenz“-Messung angegeben. In einer Publikation von Drover et al. [56] erläutert eine detaillierte Grafik die Entwicklung des Algorithmus. Spektrale, bispektrale sowie Kohärenzmessungen werden explizit aufgeführt.

White et al. [57] haben untersucht, wie sich die Indizes des PSI und BIS nach Gabe eines Propofolbolus bzw. Steigerung der Desflurankonzentration verändern, und konnten bei beiden Monitoren vergleichbare numerische Veränderungen über die Zeit erkennen. Soehle et al. [17] untersuchten 2008 den Sedline®- und BIS™-Monitor während Sevoflurananästhesien bei jüngeren Patienten (Durchschnittsalter 37 Jahre) und waren damit die ersten, die den PSI mit berechneten Wirkortkonzentrationen verglichen. Es konnte eine signifikante Korrelation zwischen PSI- und BIS-Werten beobachtet werden (r^2^ = 0,75), wobei Schneider et al. [58] eine weitaus schwächere Korrelation beobachtete (r^2^ = 0,445). Aufgrund der geschützten Algorithmen der einzelnen Indizes bleibt die Ursache dieser Unterschiede reine Spekulation. Die PSI-Werte waren bei vergleichbaren Anästhesiestadien jedoch jeweils 10 bis 15 Einheiten tiefer als diejenigen des BIS, was den unterschiedlichen publizierten Zielbereichen entspricht. PSI-Werte bei wachen Patienten liegen je nach Studie zwischen 88 und 94 [59] bzw. um 80 [60].

Der PSI, ebenso wie der BIS-Algorithmus, scheint nicht in der Lage zu sein, zuverlässig ein Wiedererlangen des Bewusstseins zu detektieren, wobei wir zwischenzeitlich wissen, dass ein provozierter Händedruck eines Patienten während einer Allgemeinanästhesie als Messinstrument nicht gleichzusetzen ist mit intraoperativer Awareness bzw. Wiedererlangen von Bewusstsein, denn hinterher können sich viele Patienten nicht mehr an den Händedruck erinnern [61, 62].

2017 haben Muhlhofer et al. [63] erkannt, dass die Detektion von Suppression durch den Sedline® Monitor weniger Episoden erkennt, als wenn ein Neurologe das EEG begutachtet. Interessanterweise war die Anzahl an Suppressionen, welche sich aus der Begutachtung durch die Neurologen ergab, mit erhöhtem Risiko für ein postoperatives Delir assoziiert, nicht aber die vom PSI erkannten. Dies lässt auf eine verminderte Sensitivität der *Burst-Suppression-Detektion* durch den PSI-Algorithmus schließen. Drover zeigte, dass mit dem Gebrauch des PSI weniger Propofol verwendet wurde, und dass die Aufwachzeiten kürzer waren als bei nicht-EEG-kontrollierten Anästhesien [56].

### Entropie-EEG-Modul (GE Healthcare™, Chicago, IL, USA)

**Figure 6:**
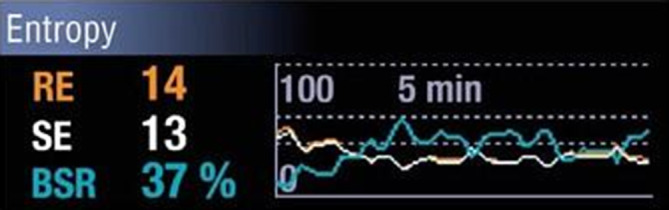

Die Beurteilung der spektralen Entropie, also der spektralen Unordnung des EEG, ist seit 2004 kommerziell als Modul für den Anästhesiemonitor verfügbar (Abb. 6). Für die EEG-Ableitung wird ein gerätespezifisches Einwegelektrodenband unilateral auf die Stirn geklebt. Das Modul zeigt 2 Indizes an. Zum einen die *Response-Entropie* (RE), welche die spektrale Entropie über einen großen Frequenzbereich (0,8–47 Hz) charakterisiert und so vermehrt auf die Aktivierung der Gesichtsmuskulatur reagiert und eine kurze Reaktionszeit unter 2 s hat. Zum anderen die *State-Entropie (SE)*, die die spektrale Entropie in den tieferen, für die anästhetikavermittelte Wirkung essenzielleren Frequenzbereichen (0,8–32 Hz) misst. Sie ist immer kleiner oder gleich der RE und reagiert langsamer auf Veränderungen. Auf PubMed finden sich 36 Publikationen zur RE und 104 zur SE. In einer Studie schneidet das Entropie-Monitoring im Hinblick auf *Kosteneffizienz* besser ab als das BIS-Monitoring, aber schlechter als der Narcotrend-Monitor [29]. Dabei muss beachtet werden, dass die Kosten und damit auch die Kosteneffizienz abhängig vom Land, von der Klinikgröße und den entsprechenden Lieferverträgen erheblich variieren kann (Tab. 1).

Der zugrunde liegende Algorithmus der Entropie ist im Vergleich zu anderen Methoden das am besten beschriebene pEEG-Monitoring-Verfahren [13]. Kurz zusammengefasst, ist die Entropie ein Maß für die Unordnung, Unruhe oder Komplexität eines Signals. Die hohe Entropie im EEG bei wachen Personen spiegelt die komplexe neuronale Informationsverarbeitung wider. Diese Bandbreite an Möglichkeiten nimmt mit zunehmendem Bewusstseinsverlust ab: Weniger Information wird vom Gehirn verarbeitet; die kortikale Entropie sinkt [13, 64, 65].

Für Sevofluran konnte eine signifikante Korrelation zwischen Entropie und der Wirkortkonzentration des Anästhetikums wie für das BIS-Monitoring gezeigt werden [66]. Für die TIVA mit Propofol scheint dagegen das BIS-Monitoring besser abzuschneiden als das Entropie-Monitoring [67]. Bezüglich der Aufwachreaktion reagiert die Response-Entropie deutlich rascher als die State-Entropie und der BIS-Index [68]. Der *Verbrauch an Propofol bzw. Sevofluran* konnte unter dem Einsatz des Entropiemodules deutlich reduziert und die *Zeit bis zur Verlegung in den Aufwachraum* (nach Allgemeinanästhesie mit Propofol/Lachgas/Alfentanil) ebenfalls verkürzt werden [69–71]. Betreffend intraoperativer Awareness, postoperativem Delir und Mortalität gibt es bisher keine größeren Studien unter Verwendung des Entropiemodules.

Die SE-Werte scheinen insbesondere in den *„Übergangsphasen“ der Anästhesie* fehleranfälliger zu sein als der BIS-Wert [72]. Dies wird von den Ergebnissen mehrerer Studien unterstrichen, die feststellten, dass zusätzlich zum SE-Wert immer das Roh-EEG betrachtet werden soll, um Fehlinterpretationen zu vermeiden [73, 74]. Die RE-Werte werden durch die Gabe von Rocuronium signifikant beeinflusst und sind daher zur Messung der Nozizeption unter Einsatz von neuromuskulär blockierenden Substanzen ungeeignet. Auch dies unterstreicht die Notwendigkeit der Beurteilung des Roh-EEG [75]. Insbesondere bei Betrachtung der SE-Werte findet man häufig kurze Episoden von Werten > 70 trotz adäquater Anästhetikakonzentration (in 3,6 % der Patienten Episoden > 2 min) [76]. Dies ist deutlich häufiger als beispielsweise beim BIS-Monitoring (0,24 %). Die Übereinstimmung zwischen dem SE- und dem BIS-Wert ist nur moderat und nimmt mit zunehmendem Alter ab. Zudem hat die Elektrodenposition einen signifikanten Einfluss auf die Werte [77]. Meybohm et al. zeigten, dass BIS- und Entropiewerte während einer Normothermie gut übereinstimmen, es aber v. a. während einer Hypothermie zu Diskrepanzen kommt. Dies muss klinisch v. a. bei Herzoperationen in Hypothermie berücksichtigt werden [78]. Eine Studie konnte nachweisen, dass bei 4 % der Patienten der zeitglich links- und rechtshemisphärisch gemessenen SE-Wert um mehr als 10 Punkte differiert. Der SE-Wert sollte daher nicht zur alleinigen Beurteilung der „Anästhesietiefe“ herangezogen werden.

### qCON (Conox, Quantium Medical, Mataró, Spanien)

**Figure 7:**
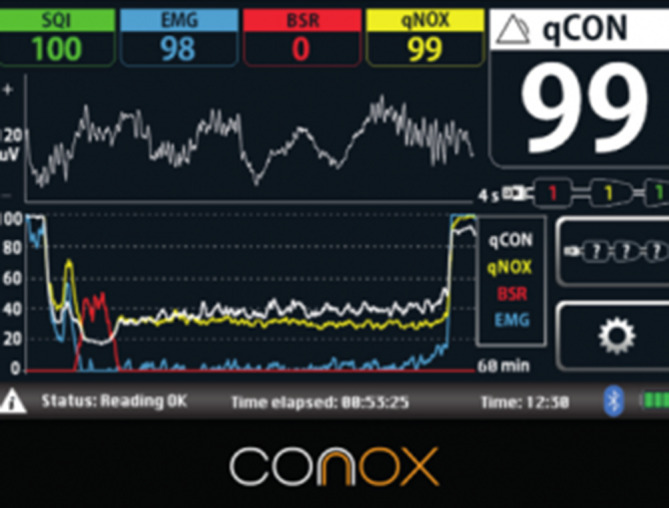

Der Conox Monitor der Fa. Quantium Medical (Abb. 7) ist seit 2013 auf dem Markt erhältlich. Mittels frontal angebrachtem, gerätespezifischem Einwegelektrodenband berechnet er aus einem EEG-Kanal den qCON-Index (für „consciousness“) von 0 (Koma) bis 100 (wach) als Äquivalent für die „Anästhesietiefe“. Außerdem wird in den neueren Modellen auch der sog. qNOX-Index (für „noxiousness“) berechnet, der die Wahrscheinlichkeit angibt, auf einen Schmerzreiz zu reagieren (Werte von 0 (Reaktion unwahrscheinlich) bis 99 (Reaktion wahrscheinlich)). Beide Indizes basieren auf prädiktiven Modellen, wobei für die Berechnung des qCON-Index 15 EEG-Parameter genutzt werden. Genauere Details zu diesen Parametern sind nicht beschrieben. Unter einem qCON-Wert von 25 wird der qNOX-Index nicht berechnet, da von einer ausreichenden „Anästhesietiefe“ ausgegangen werden kann.

Die Studienlage zum Conox-Monitor fällt spärlich aus. Bei PubMed finden sich mit dem Suchbegriff „qCON“ lediglich 12 Artikel zum Thema Neuromonitoring, wovon etwa die Hälfte von Personen mit einer starken Affiliation zum Hersteller Quantium Medical publiziert wurde. Der qCON-Index wurde validiert, indem EEG-Daten aus einer vorhergehenden BIS-Monitoring-Studie während einer Allgemeinanästhesie mit Propofol mit dem qCON-Algorithmus erneut berechnet und verglichen wurden [79].

Der qCON-Wert hat eine ähnliche zeitliche Verzögerung wie der BIS oder Narcotrend nach raschen Veränderungen der „Anästhesietiefe“ [80]. Der qNOX-Index hingegen zeigt das *Wiedererlangen des Bewusstseins* oder eine *Reaktion auf einen Schmerzreiz* früher (bis zu einer Minute vor Erwachen) an [81].

Es gibt bisher keine Studien zu intraoperativer Awareness und deren Vermeidung mit dem Gebrauch des Conox-Monitors. Aus diesem Grund muss unter Vorbehalt auf die entsprechenden Studien anderer pEEG-Monitore verwiesen werden.

Während Sedationen mit Propofol zeigt der Conox-Monitor im Vergleich zum BIS- und Entropiemonitor signifikant tiefere Werte an. Der Grund dafür scheint eine unterschiedliche Skalierung der Indizes zu sein, da alle 3 Monitore ähnliche Trends und Reaktionen auf Reize (Bronchoskopie, Husten) aufweisen [82]. Es gibt Hinweise dafür, dass Patienten mit höheren intraoperativen qCON-Werten einen bis zu 25 % erhöhten postoperativen Opiatbedarf haben [83]. Der qNOX-Wert kurz vor Ausleitung zeigte hingegen keinen Zusammenhang zu postoperativen Schmerzen im Aufwachraum [84]. Nach Gabe eines i.v.-Bolus Ketamin während einer Allgemeinanästhesie mit Desfluran steigt der qCON-Wert im Unterschied zu anderen Monitoren nicht signifikant an und scheint somit weniger von höheren Frequenzbereichen abhängig zu sein. Dies führt v. a. bei *multimodaler Anästhesieführung* potenziell zu stabileren Indexwerten [85].

## Das prozessierte EEG in der klinischen Anwendung

Trotz der oben bei allen Monitoring-Verfahren diskutierten, teils umstrittenen Datenlage wird die Verwendung eines „Anästhesietiefe“-Monitors zur Senkung der Inzidenz des postoperativen Delirs in Risikogruppen von einigen Fachgesellschaften empfohlen [86, 87]. So empfehlen die Deutsche Gesellschaft für Anästhesiologie und Intensivmedizin (DGAI) und die European Society of Anaesthesiology and Intensive Care (ESAIC) die Verwendung eines Neuromonitorings, sofern verfügbar und insbesondere bei Patienten mit hohem Risiko für intraoperative Awareness oder postoperatives Delirium sowie bei gleichzeitiger Verwendung von Muskelrelaxanzien (s. oben) [86, 88, 89].

## Auswahl des Monitors

Aus den Erläuterungen der unterschiedlichen pEEG-Monitore lässt sich keine evidenzbasierte Empfehlung ableiten, da alle Geräte inhärente Stärken und Schwächen aufweisen. Die unterschiedlichen Studiendesigns zu den einzelnen Monitoren lassen sich schwer miteinander vergleichen. Viele Studien nehmen Bezug auf den BIS™-Monitor, weil hier die Datenlage am breitesten ist (u. a. wegen der früheren Markteinführung). Außerdem gibt es keine Studie, die alle oben genannten Monitore miteinander vergleicht. Meistens werden 2 oder maximal 3 Geräte gegenübergestellt. Die Studienlage zu Awareness- und Delirinzidenz im perioperativen Setting ist – außer für den BIS™-Monitor – für die meisten Monitore ungenügend und auch für den BIS™-Monitor wie dargestellt kontrovers.

Zudem spielen institutionelle, finanzielle und anwendungsspezifische Faktoren für die Wahl eine große Rolle. In Tab. 1 ist eine Kostenübersicht für die verschiedenen Geräte dargestellt. Ein Qualitätsmerkmal für die Anschaffung eines EEG-Monitorings sollte allerdings sein, dass neben dem Index auch das Roh-EEG, ein Spektrogramm sowie ein Elektromyogramm (EMG) sichtbar sein sollten. Diese Darstellungsformen des (spektralen) EEG geben Veränderungen beinahe in Echtzeit wieder, im Gegensatz zu den pEEG-Monitoren, bei welchen es vom eingestellten Intervall abhängt, über welches der Indexwert berechnet und gemittelt wird, sowie davon, wie häufig ein neuer Indexwert ausgegeben wird. Beim BIS™-Monitor lässt sich das Glättungsintervall z. B. auf 10, 15 oder 30 s einstellen. Somit kommt es je nach Grundeinstellung zu unterschiedlichen Verzögerungen von 20–60 s in der Darstellung von Veränderungen im EEG [90, 91].

## Problematik der Hysterese und sigmoidalen Dosis-Wirkung-Beziehung

Der pEEG-Index verändert sich dynamisch während Induktion, Erhaltung und Ausleitung einer Allgemeinanästhesie sowie auf spezifische Ereignisse wie Schmerz, Hypoperfusion oder Boli von Medikamenten. Der Index sollte daher in groben Zügen in sinnvoller Beziehung zur Anästhetikakonzentration oder zum Ereignis stehen. Darum ist der Beginn der Messung bereits beim wachen Patienten empfehlenswert. Bereits kurz nach den Anästhesieeinleitung können v. a. bei älteren oder komorbiden Patienten rasch niedrige Indexwerte aufgrund von Burst Suppression im EEG beobachtet werden [92]. Dies hängt womöglich mit einer erhöhten Sensibilität des Gehirns für Anästhetika als auch einer langsameren Verteilung und dadurch Verzögerung des Effekts auf das Gehirn zusammen [93, 94]. Die Indexwerte kehren in der Regel durch Anpassung der Dosis des Anästhetikums rasch in den vom Hersteller empfohlenen Bereich zurück. Nach Abstellen des Anästhetikums am Ende der Operation zeichnet sich wiederum verzögert die Ausleitung im prozessierten EEG-Index ab. Dabei bleiben die Indexwerte im klinischen Setting in der Regel unter den vor der Einleitung abgelesenen „Wachwerten“ (Hysterese von Anästhetika) [95].

Aufgrund der sigmoidalen Beziehung (Abb. 1) zwischen pEEG-Index und der Anästhetikakonzentration im Gehirn kann es im Bereich des Verlustes sowie beim Wiedererlangen des Bewusstseins zu abrupten Änderungen des Index bei nur geringer Veränderung der Anästhetikakonzentration kommen. Diese rasche Dynamik muss insbesondere während des Versuchs der Titration der Anästhetika bei komorbiden oder hämodynamisch instabilen Patienten beachtet werden, da es hierbei eher zu akzidentieller intraoperativer Awareness kommen könnte, bei gleichzeitig aufgehobener Reaktion des vegetativen Nervensystems.

## Troubleshooting unklarer bzw. unerwarteter „Anästhesietiefe“-Indizes

**Figure 8:**
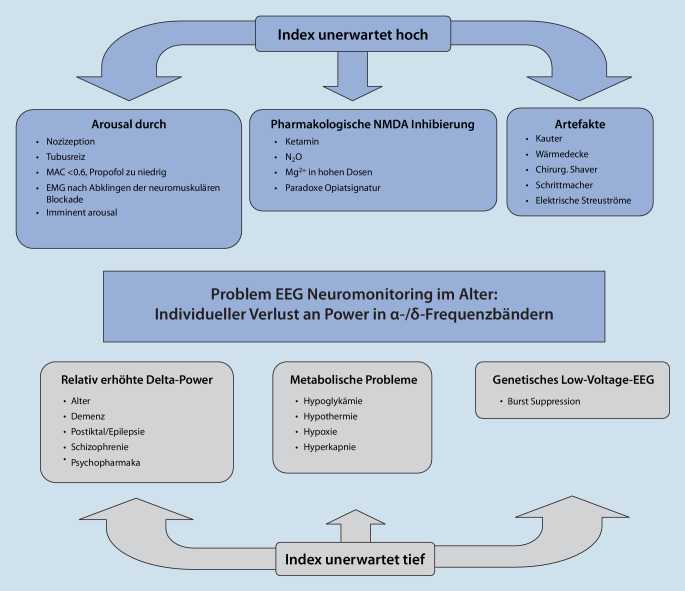

In Abb. 8 sind die häufigsten Ursachen für falsch-hohe oder tiefe pEEG-Indizes dargestellt. Steigt der pEEG-Index plötzlich und unerwartet an, gilt es zunächst, unbeabsichtigtes Erwachen aufgrund inadäquater Anästhetikadosierung oder Analgesie auszuschließen. Hierfür sind zumindest grundlegende Kenntnisse des Roh-EEG hilfreich. Bei nichtmuskelrelaxierten Patienten kann das EMG, welches sehr sensibel auf kleinste Gesichtsmuskelaktivität reagiert, wegweisend sein. Pragmatischerweise begegnen Kliniker dieser Situation häufig mit gleichzeitiger Gabe eines Analgetikums und Vertiefung der Anästhesie [96].

Ist die Wahrscheinlichkeit eines nozizeptionsbedingten Erwachens gering, so müssen im nächsten Schritt medikamentöse Ursachen für einen angestiegenen Index ausgeschlossen werden. Ein wichtiges Beispiel ist die Gabe von N‑Methyl-D-Aspartat(NMDA)-Rezeptor-Antagonisten (Ketamin, Lachgas, Magnesium). Äußerst selten kann der pEEG-Index auch paradox auf eine Opiatgabe ansteigen, da die Beta-Aktivität im perioperativen EEG z. B. durch NMDA-wirksame Opiate (Remifentanil, Methadon etc.) ansteigen kann [97]. Sehr häufig steigen pEEG-Werte außerdem aufgrund von Artefakten an. So können z. B. Elektrokauter, Wärmedecken, oszillierende chirurgische Shaver (z. B. für Arthroskopien) oder kardiales Overdrive Pacing die mögliche Ursache darstellen.

Sinken die pEEG-Indizes auf unerwartet tiefe Werte, können neben einer Überdosierung der Anästhetika auch ein kürzlich verabreichtes Muskelrelaxans, ein eben applizierter Bolus eines Opioids oder die Verwendung von Psychopharmaka (inklusive Benzodiazepinen zur präoperativen Anxiolyse) ursächlich sein. Paradoxerweise kann auch ein Schmerzreiz durch einen abrupten Verlust von frontalen Alpha-Oszillationen zum plötzlichen Absinken der pEEG-Indizes führen [98]. Außerdem können kritische Veränderungen der Körpertemperatur [99], der Hirndurchblutung und der Homöostase von CO_2_ oder Blutzucker zu deutlichen Abfällen der pEEG-Indizes führen. Sehr alte oder demente Patienten können von Beginn an sehr tiefe pEEG-Indizes aufweisen, da je nach Monitor die für den pEEG-Algorithmus wichtigen Frequenzbereiche und Oszillationen nur noch schwach oder atypisch ausgebildet sind. Ähnlich verhält es sich bei postiktalen Patienten. Epileptische Potenziale im EEG können sowohl ein Ansteigen (hohe Frequenz des rhythmischen EEG) als auch deutliches Abfallen der pEEG-Indizes (postiktal, hohe Synchronizität während Anfall) bewirken. Bei pathologischem Alkoholkonsum sind paradoxe Reaktionen auf Propofol beschrieben [100].

## Einbeziehen weiterer Parameter für die Einschätzung der „Anästhesietiefe“

Das frontale EEG repräsentiert die Aktivität im kortikothalamischen System und eignet sich darum zur Einschätzung der „Anästhesietiefe“. Daneben gibt es aber klinische Zeichen, deren Beurteilung in der Abschätzung der „Anästhesietiefe“ nützlich sein können. Hierzu zählen die Hirnstammreflexe [96], wie Korneal- und Lidreflex, und die Reaktion des vegetativen Nervensystems auf Stress mit Anstieg von Herzfrequenz und Blutdruck, Pupillenerweiterung und Bronchokonstriktion (frühzeitig erkennbar in der Druck-Volumen- oder Fluss-Volumen-Kurve am Beatmungsgerät). Keine dieser einzelnen Testmethoden garantiert bei wechselnder Intensität chirurgischer Schmerzreize eine adäquate Messung der „Anästhesietiefe“. Zusammen ergeben sie jedoch ein immer dichteres diagnostisches Netz. Das EEG spielt dabei eine wichtige, zusätzliche Rolle zur Detektion einer zu tiefen und nicht nur einer zu oberflächlichen Allgemeinanästhesie.

Insbesondere im Licht neuer Anästhetikakombinationen, wie sie in der *multimodalen Anästhesie* von Gesellschaften wie der Enhancend Recovery After Surgery (ERAS) Society gefordert werden [102], gewinnt die EEG-Diagnostik an Bedeutung. Klinische Befunde wie Hirnstammreflexe bei nichtmuskelrelaxierten Patienten können zur Beantwortung der Frage „Ist die Analgosedation dieser Anästhesie ausreichend?“ herangezogen werden. Die Gabe von Ketamin und Magnesium sowie die Kombination opiatsparender Allgemeinanästhesie mit Regionalanästhesien führen teilweise zu Indexwerten des pEEG außerhalb der empfohlenen Bereiche für eine Allgemeinanästhesie und im quantitativen EEG für viele Kliniker zu unbekannten EEG-Signaturen [103]. Dies zeigt klar die Grenzen der pEEG-gesteuerten Anästhesie auf. Daher sollten die Kennsignaturen typischer Anästhetikakombinationen im quantitativen EEG (hier v. a. im Spektrogramm) – wie sie im Teil 1 dieses Artikels dargestellt werden – allen Anästhesisten bekannt sein [1].

## Diskussion

Wie aus der Vorstellung der vorhandenen pEEG-Algorithmen ersichtlich ist, befindet sich das Neuromonitoring gegenwärtig an einem wichtigen Scheideweg. Die hohen Versprechen, mit denen prozessierte EEG-Indizes eingeführt wurden, konnten in der alltäglichen Praxis nicht nachhaltig erfüllt werden [32, 39]. Darüber hinaus sind – wie oben detailliert beschrieben – lediglich für die Verwendung des BIS™-Monitors Daten zur intraoperativen Awareness sowie zum postoperativen Delir und Mortalität vorhanden. Diese dürfen aufgrund der unterschiedlichen Algorithmen nicht unreflektiert auf andere pEEG-Monitore übertragen werden.

Zudem muss bedacht werden, dass die Definition einer optimalen pEEG-basierten „Anästhesietiefe“ schwierig ist. Insbesondere alte und komorbide Patienten sind vermutlich im Rahmen fortgeschrittener Hirnatrophie nicht in der Lage, die zugrunde liegenden kortikothalamischen Kennsignaturen im Schlaf oder der Anästhesie deutlich herauszubilden. Inwieweit diese fehlende Alpha-Signatur, die in den Spektrogrammen offensichtlich ist, die pEEG-Indizes beeinflusst, ist unklar [101]. Festzustellen ist ebenfalls, dass der BIS-Index, die State-Entropie sowie der qCON im Gegensatz zum Narcotrend ca. 0,2 Indexpunkte/Lebensjahr oder 2 Punkte/Lebensdekade bei einem bestimmten altersadaptierten MAC-Wert ansteigen. Inwieweit dies aber eine klinische Relevanz darstellt, bleibt fraglich, wenn man bedenkt, dass ein Zielbereich von 20 Punkten Unterschied (meist ein Index zwischen 40 bis 60) anvisiert wird und die individuelle Variabilität bei einem bestimmten altersadaptierten MAC-Wert bis zu ± 7 Indexpunkte beträgt [51, 52, 104]. Ein wesentliches Problem solcher Vergleiche ist, dass diese gegen den altersadaptierten MAC-Wert erfolgten und dieser auf einem standardisierten Schmerzreiz beruht und keinem EEG-Korrelat des Großhirns [105].

PEEG-Indizes bieten vielen Anästhesisten eine „Scheinsicherheit“. Der Ansatz, dieses komplexe nichtlineare System der Bewusstseinsreduktion auf einer Skala von 0–100 darzustellen, erscheint gewagt und verleitet den Anwender indirekt dazu eine 10 %ige Reduktion auf der Skala mit einer 10 %igen Zunahme der Anästhesietiefe gleichzusetzen [23]. Es hat sich gezeigt, dass keiner der aufgeführten pEEG-Monitore ein intraoperatives, dem Erwachen identisches EEG-Muster, verlässlich einordnen kann [106]. Demzufolge scheinen ein Umdenken sowie die Ausbildung von Anästhesisten in Roh-EEG Signalen unabdingbar.

Wenn gesteigerte Aktivität der Arousal-Systeme bei Nozizeption das Erwachen aus der Anästhesie einleiten, könnte die zeitliche Latenz bis zur Abbildung im pEEG-Algorithmus (Abb. 8) relevant sein. Eine Eingrenzung des Zeitrahmens, wie rasch intraoperative Awareness entstehen kann, wäre momentan aber rein hypothetisch. Zudem stellt die Neurobiologie die führende Rolle des Kortex für das „wache Bewusstsein“ infrage [107, 108]. Insbesondere Schmerz und Wachheit sind von der Aktivität mehrerer zerebrospinaler Netzwerke abhängig, deren Gewichtung im „kortikozentrischen“ Modell verloren geht. Vermehrte Aufmerksamkeit in der Auswertung der Aktivität des Hirnstammes und des vegetativen Nervensystems könnten in Kombination mit einer EEG-Analyse zu einem besseren Monitoring des gesamten Hirnes führen.

Ein weiterer Nachteil bei der Fokussierung auf pEEG-Indizes ist, dass die eigentliche Auswertung physiologischer Hirnsignale (wie z. B. intraoperativer Alpha-Oszillationen), die in direktem Zusammenhang mit klinischen Endpunkten wie postoperativer Analgesie oder kognitiver Integrität stehen, unterbleibt [109, 110].

Unter Allgemeinanästhesie kommt es zu einer typischen Abnahme der Konnektivität zwischen Hirnregionen [111–113]. Neben der rein frontalen EEG-Ableitung für die Generierung der derzeitigen „Anästhesietiefe“ könnten wohl zusätzlich okzipitale Kortexregionen für die Beurteilung von Veränderungen des Bewusstseins genutzt werden [114, 115]. Die Messung solcher dynamischen neurobiologischen Prozesse wie der Alpha-Anteriorisierung unter Narkose findet aber v. a. aus Gründen der Praktikabilität noch keine breite klinische Anwendung [115–117].

## Fallvignette, Teil 2

**Figure 9:**
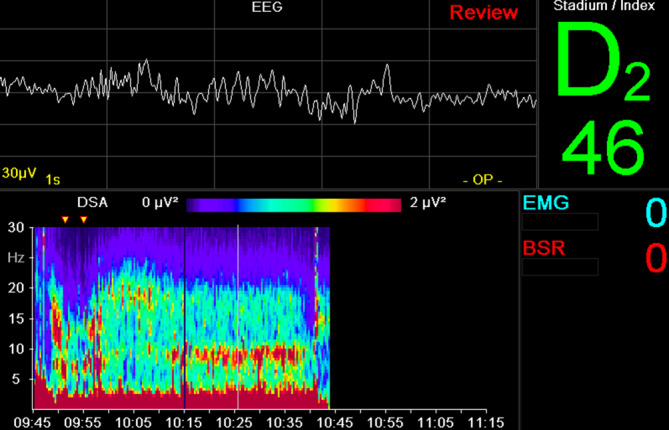

Anhand des ausgewählten komplexen Fallbeispieles soll nun die alltägliche Problematik in der Anwendung der derzeitigen pEEG-Monitore aufgezeigt und näher erläutert werden. Die kurze Anästhesiedauer und das geringe Ausmaß des chirurgischen Eingriffes haben sicher zu einem komplikationslosen Verlauf beigetragen. In der Vorgeschichte waren jedoch schwere Delirverläufe auch bei kleineren endoskopischen Interventionen unter Propofolsedation beschrieben. Es ist anzunehmen, dass nebst der multimodalen pharmakologischen Intervention (Clonidin- und Melatoninprämedikation, Dexmedetomidin- und Ketaminbolus, TIVA mit Propofol) die Kombination mit einem Subtenonblock den erfreulichen delirfreien Verlauf ermöglicht hat [86, 102, 118]. Der multimodale pharmakologische Ansatz führt aber auch zu spezifischen Veränderungen des perioperativen EEG. Hierbei fällt ab 09:58 sowohl ein zeitweise nichtberechenbarer bzw. fehlender pEEG-Index als auch im weiteren Verlauf ansteigender und hoher (> 64) Narcotrend-Index auf (Abb. 2). Dies könnte ohne Interpretation des quantitativen EEG (Spektrogramm) als Zeichen einer inadäquaten und zu oberflächlichen „Anästhesietiefe“ gewertet werden (Abb. 9) und Anästhesisten dazu verleiten, die Propofoldosis zu erhöhen. Bei Betrachtung des Spektrogramms (Abb. 9) fällt jedoch auf, dass sich unmittelbar nach der Ketaminapplikation, die nach der Einleitung kurzfristig vorhandene Alpha/Delta-Aktivität (09:55–09:58) in eine Beta/Delta-Aktivität verändert (ab 10:00). Ketamin führt über die Inaktivierung des NMDA-Rezeptors zu einer kortikalen Enthemmung, was im EEG als vermehrte Beta-Aktivität gekennzeichnet ist [1]. Dies könnte neben induzierten Artefakten während der Durchführung des Subtenonblocks (10:00–10:10) ein Grund für die nichtberechneten und später erhöhten Indexwerte sein. Die intensiven Alpha-Oszillationen zwischen 8 und 10 Hz ab 10:15 (rotes horizontales Band im DSA) entsprechen – wie die Aufnahme des Roh-EEG zeigt – zum großen Teil den dicht aufeinanderfolgenden „Schlafspindeln“. Diese „Alpha-Oszillationen“ sind eingebettet in eine kräftige Beta-Grundaktivität, die für den Anstieg des pEEG verantwortlich sein dürfte. Das Auftreten solcher anästhesietypischer Spindeln oder Oszillationen im Alpha-Frequenzbereich stellt ein physiologisches Signal dar, das auf die Abkoppelung der kortikothalamischen Bewusstseinszentren von der Peripherie des Nervensystems und somit eine ausreichende „Anästhesietiefe“ hinweist. Solche wertvollen Informationen gehen bei alleiniger Betrachtung des pEEG verloren. Da das Auftreten dieser „Alpha-Oszillationen“ während einer Allgemeinanästhesie mit einer Reduktion des unmittelbar postoperativen Delirs und evtl. einer höheren Wahrscheinlichkeit guter perioperativer Analgesie vergesellschaftet, bietet vielleicht eher das Roh-EEG als das pEEG das Potenzial zur Verbesserung des Outcomes unserer Patienten [109].

## Fazit für die Praxis

- Eine klare Empfehlung für ein bestimmtes prozessiertes EEG-Monitoring kann aufgrund der Studienlage nicht gegeben werden.
- Das prozessierte EEG liefert nur eingeschränkt Hilfe zur Interpretation der „Anästhesietiefe“. Es sollten daher unbedingt weitere Modalitäten wie vegetative Symptome, Pupillendurchmesser, Hirnstammreflexe und quantitatives EEG miteinbezogen werden!
- Nicht verwertbar zur Interpretation der „Anästhesietiefe“ sind prozessierte EEG-Indizes bei einem opiatfreien, multimodalen Anästhesieansatz speziell bei Verwendung von Ketamin.
- Die höchste Inzidenz für intraoperative Awareness in Anbetracht der Wahl des Hypnotikums besteht:
  – während einer TIVA (v. a. in Kombination mit einer Muskelrelaxation) ohne Nutzung eines prozessierten EEG-Monitors,
  – während einer volatilen Allgemeinanästhesie ohne Alarm für eine untere Grenze für die endtidale minimale alveoläre Konzentration.
- Alle größeren Outcome-Studien zu intraoperativer Awareness, postoperativem Delirium und Mortalität wurden bisher mit dem BIS™-Monitor durchgeführt. Die Ergebnisse sollten aber aufgrund der Unterschiede im Algorithmus der verschiedenen Monitore nicht auf alle prozessierten EEG-Monitore generalisiert werden.
- Im „Troubleshooting“ unklarer pEEG-Indizes gilt es, zügig die gravierendsten Ursachen (akute Nozizeption im Kontext des operativen Eingriffs, Erwachen, hämodynamische Instabilität etc.) auszuschließen und zu behandeln.

## Footnotes

1 Wegen unterschiedlicher Definitionen der Anästhesietiefe und Hinweisen, dass es sich dabei um ein multidimensionales Konzept handelt, setzen wir in diesem Artikel die Anästhesietiefe in Anführungszeichen.
